# Spectral and temporal differentiation between integral and contaminant chlorophyll *a* in the cytochrome *b*_6_*f* complex

**DOI:** 10.1039/d5cp03433g

**Published:** 2025-12-02

**Authors:** Adrien A. P. Chauvet, Rachna Agarwal, William A. Cramer

**Affiliations:** a Laboratoire de Spectroscopie Ultrarapide (LSU), ISIC, Faculté des Sciences de Base and Lausanne Centre for Ultrafast Science (LACUS), Ecole Polytechnique Fédérale de Lausanne (EPFL) Station 6 1015 Lausanne Switzerland a.chauvet@sheffield.ac.uk; b Department of Biological Sciences, Purdue University West Lafayette Indiana 47907 USA; c Molecular Biology Division, Bhabha Atomic Research Centre Mumbai 400 085 India

## Abstract

Purification of photosynthetic protein complexes in detergent often results in residual contaminant chlorophyll (Chl) that is dependent on the preparation process. In the case of the cytochrome *b*_6_*f* complex, which contains one molecule of bound Chl *a* per 130 kDa monomer in the dimeric hetero-oligomeric complex, both complex-bound and contaminant Chl *a* are present and spectrally indistinguishable in the steady-state absorbance spectra commonly employed to assay photosynthetic protein complexes. We, however, demonstrate that the signals from photo-excited cyt *b*_6_*f*-bound and contaminant Chl *a* have distinct temporal and spectral signatures, as revealed by ultrafast optical transient spectroscopy. The difference in signals is further amplified by using a non-anisotropic pump–probe scheme to enhance the detection of the contaminant Chl *a* stimulated emission. Such sharp differences further exemplify the impact of the environment on the photodynamic properties of molecules.

## Abstract 2

Ultrafast transient spectroscopy enables disambiguation between integral and contaminant chlorophyll (Chl) in photosynthetic protein complexes.

## Graphical abstract

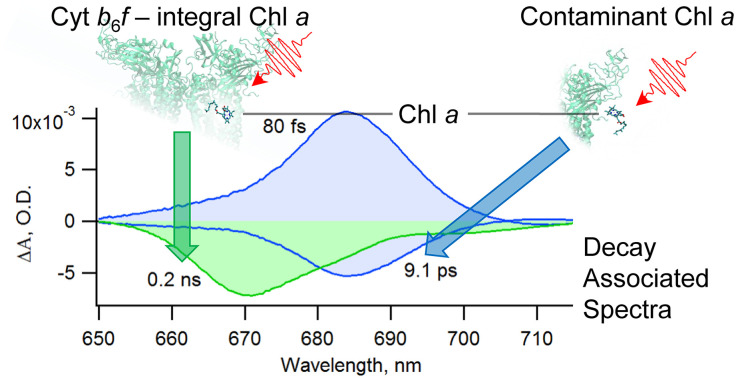

## Introduction

**Fig. 1:**
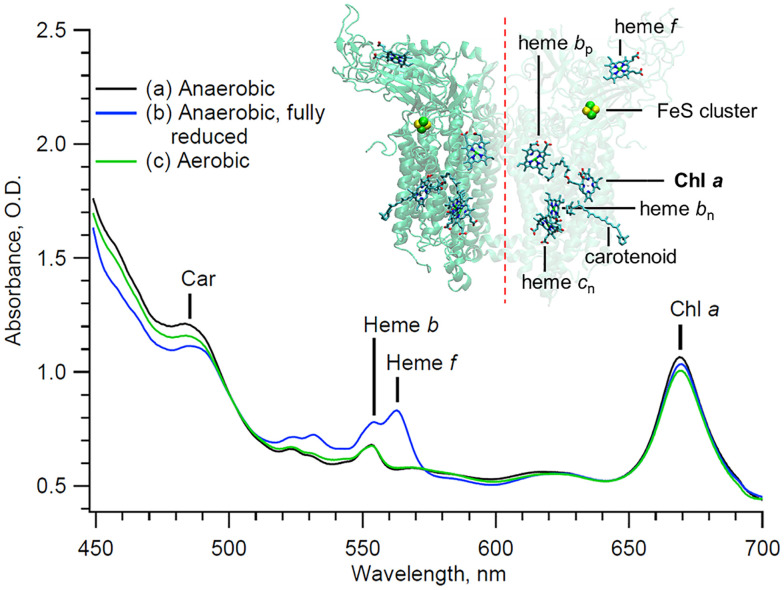
Steady state of the cytochrome *b*_6_*f* complex at different redox states and under different conditions (see text). Inset: Backbone structure and prosthetic groups of the cytochrome *b*_6_*f* complex. The crystallographic data are taken from the 2E74 pdb file.^7^

The study of interacting pigments, especially in the case of photosynthetic protein complexes, provides valuable information about quantum coherence, energy transfer, and electronic conformation of the proteins involved.^1,2^ However, ultrafast spectral analyses are highly sensitive to the sample quality, as any contaminant affects the signal and its resolution. In the case of the multi-heme cytochrome (cyt) *b*_6_*f* complex,^3^ solubilized chlorophyll (Chl) *a* often remains in the sample even after isolation and purification in detergent. Unfortunately, this Chl *a* contaminant is spectrally indistinguishable in static measurements compared to the single native embedded Chl *a* molecule (Fig. 1).^4,5^ The Chl *a* purity of these samples is therefore challenging to assess using standard steady state techniques. Although high-quality cyt *b*_6_*f* has been obtained, leading to the crystallographic resolution of the protein complex,^6^ the extent of Chl contamination in the purified complex remains a problem, and an efficient and quantitative assay would facilitate structural studies as well as studies of the dynamics of the cytochrome complex.^6^

Current optical methods for assessing the Chl *a*/cyt *f* ratio are based on the evaluation of the intensity of the sodium dodecyl sulfate (SDS) gel marker or on the absorbance ratio of cyt *f* to Chl *a*, which depends on the extinction coefficient used.^8,9^ Alternatively, Savikhin *et al.* showed that impure cyt *b*_6_*f* preparations were marked by an additional short ∼ps lifetime, which was then ascribed to contaminant Chl *a*.^4^ This work pushes the disambiguation one step further and demonstrates that it is possible to also spectrally distinguish between non-essential contaminant and integral Chl *a* using ultrafast transient analysis. We show that each Chl *a* type has distinct spectral and temporal signatures.

## Methods

The cytochrome b_6_f sample was prepared as follows: active dimeric cytochrome *b*_6_*f* complexes were isolated from the leaves of *Spinacia* as previously described.^10^ The subunit composition of the *b*_6_*f* preparation was assessed by using SDS polyacrylamide gel electrophoresis (PAGE), clear-native PAGE, and the redox difference spectra using standard procedures.^11^ All assays were performed in 30 mM tris(hydroxymethyl)aminomethane (Tris)-HCl (pH 7.5), 50 mM NaCl, 0.2 mM ethylenediaminetetraacetic acid (EDTA), and 0.04% undecyl maltoside (UDM). The electron transport activity of the dimeric complex, 150–200 electrons per cyt *f* s, was assessed using decyl-plastoquinol as an electron donor and *Chlamydomonas* plastocyanin as an electron acceptor.^11^ All free-floating Chl *a* contaminant molecules were effectively washed out during the isolation and purification process. Only Chl *a* molecules that are integral to the protein complex, as well as Chl *a* molecules that are non-selectively adsorbed onto the protein complex, remain. The sample, consisting of about 300 µL, was housed in a microfluidic flow-cell.^12^ The cyt *b*_6_*f* complex was studied under a controlled atmosphere using a bespoke miniature anaerobic chamber (1L).^13^ The integrity of the complexes over the course of the experiment is directly monitored by recording their steady-state absorbance through the white light continuum of the probe beam. From the steady state absorbance difference spectra, it can be inferred that the preparation contains a Chl *a* : cyt *f* ratio of approximately 1.3 : 1. The concentration of the sample is about 50 µM for the Cyt *b*_6_*f* dimer.

Transient absorption signals were recorded as follows: the 800-nm output of a 1 kHz regenerative amplifier is used to pump a home-made visible non-collinear optical parametric amplifier (NOPA, see ref. 14 for a detailed description) producing ∼40 fs, 515 nm pump pulses with a full-width-half-maximum of 15 nm. By centering the pump at 515 nm, the goal is to minimize exciting carotenoid (Car) and the various hemes, while avoiding the chlorophyll (Chl) *a Q*_*x*−*y*_ regions, as depicted in Fig. S1 (see the SI). The undisturbed 600–650 region is thus solely characterized by the signal from Car, which can then be modelled. We take advantage of the lack of sharp Car features in the Chl *a Q*_*y*_ region to estimate the Car signal as a first order polynomial and subtract it for each time delay and extract the sole Chl *a* signal, as discussed later. A small fraction of the regenerative amplifier output is focused onto a 5-mm thick CaF_2_ crystal to provide a broad visible probe. The resulting pump–probe cross-correlation signal is about 150 fs. The relative polarization between the pump and probe beam is either set to the magic angle (54.7°) or parallel in order to enhance the detection of the Chl *a* signals,^15^ as discussed in the SI. The probe beam is then focused onto a spectrometer, resulting in a probe window extending from 350 nm to 750 nm with a spectral resolution of 1.3 nm.^16^ The average excitation power was kept low, within the linear excitation regime (checked *via* power dependence), at 0.2 mW and focused on a spot size of 100 × 160 µm. Considering 40 fs-long pulses at 1 kHz, we estimate the energy to be about 200 nJ per pulse, 10% of which is lost on passing through the anaerobic chamber's windows. We thus expect the excitation energy to be about 180 nJ per pulse at the sample stage. The estimated proportion of the excited molecules is estimated to be less than 2% based on the direct comparison between the sample's absorbance (∼0.6 OD for Chl *a Q*_*y*_ absorption) and the maximum transient signal amplitude (∼0.01 OD for Chl *a Q*_*y*_ signal).

## Results and discussion

**Fig. 2:**
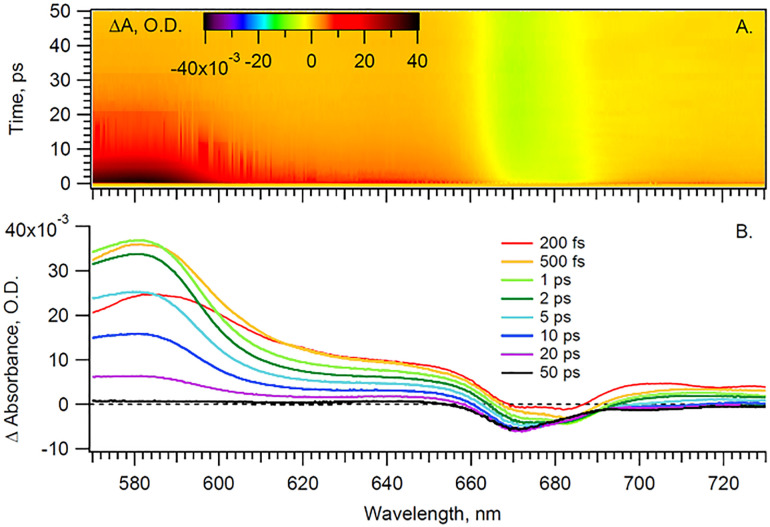
(A) Time-wavelength surface and (B) spectra at given time delays for Cyt *b*_6_*f* under anaerobic conditions and parallel pump–probe polarization.

With the pump beam centered at 515 nm, both Chl *a* and β-carotene (Car) are directly excited. Accordingly, the transient optical signal is dominated by the Car and Chl *a* responses in the 570 to 730 nm region (Fig. 2). The choice of parallel polarization between the pump and the probe significantly enhances the SE signal detection over other transient absorption signals, and without affecting the motored dynamics of Chl *a*. Indeed, the anisotropy of the cyt *b*_6_*f*-bound Chl *a*, measured in the *Q*_*y*_-band, was found to be close to the theoretical maximum of 0.4 and nearly constant over the first 200 ps.^4^ The transient signal of solubilized Chl *a* was also shown to be constant at least during the first 5 ps after excitation.^17,18^ A constant anisotropy indicates that the parallel and perpendicular polarization signals have the same dynamics as the isotropic signal monitored at the magic angle. The signal monitored here can therefore be directly compared to usual isotropic studies found in the literature, at least within these timescales. Furthermore, the Chl *a Q*_*y*_ signal, in the 650–710 nm region, is assumed to be free of any spectral contribution from the hemes *f* and *b*, which have their α-absorbance bands at 554–563 nm (Fig. 1). The carotenoid signal, on the other hand, has a ‘red tail’ that extends from 600 to 750 nm and beyond. This signal is characteristic of the optically forbidden S_1_ state of Car, which is populated *via* vibrational relaxation of the excited S_2_ at 515 nm.^19^

**Fig. 3:**
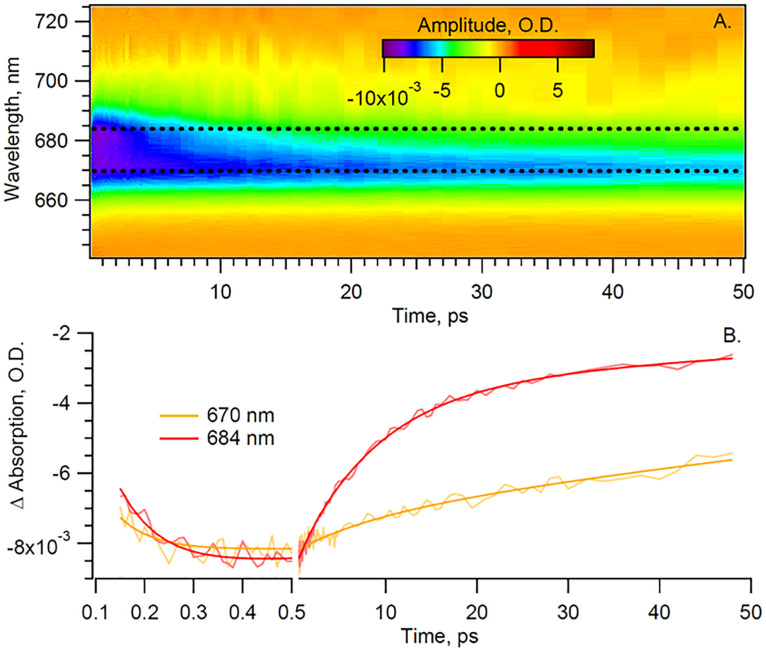
(A) The retrieved Chl *a* signal and (B) kinetics of the sole Chl *a* signal from Cyt *b*_6_*f* under anaerobic conditions and parallel pump–probe polarization. The smoothed lines represent fits of the data.

In order to disentangle the Chl *a* signal from the extended red-tail signal from Car, we take advantage of the lack of sharp features of the latter in the Chl *a Q*_*y*_ region and model the Car signal as a first-order polynomial. This background is then evaluated and subtracted for each time delay, thus enabling us to extract the sole Chl *a* signal shown in Fig. 3 heat map.

**Fig. 4:**
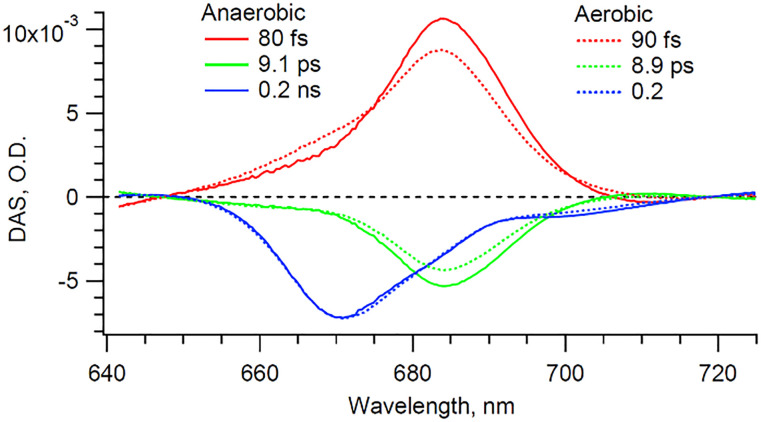
DAS of the retrieved Chl *a* signals under anaerobic and aerobic conditions.

The extracted Chl *a* signal, shown in Fig. 3A, shows two bands peaking at 670 and 684 nm. We take as a representative of each of the two bands the kinetics at 670 and 684 nm (Fig. 3B). The extracted Chl *a* signal is analyzed by means of global fit (GF), to estimate the number of exponential components, and singular value decomposition (SVD), to refine the decay times. The resulting decay-associated spectrum (DAS) is shown in Fig. 4.

The extracted anaerobic Chl *a* signal is satisfactorily fit with a minimum of three exponential decay components: (1) a ∼80 fs DAS component corresponding to the rise of a band maximizing at 684 nm. This component corresponds to the growth of a negative transient absorption signal and is assigned to either the photobleaching (PB) or the SE signal from a pool of Chl *a* molecules. However, since no specific band is seen near 684 nm in the cyt *b*_6_*f* static spectrum, the signal is solely attributed to SE. (2) A ∼9 ps DAS component, which mirrors the previous ∼80 fs DAS. Accordingly this ∼9 ps component is assigned to the decay of the state from which the SE originates; in agreement with a previous report on solubilized Chl *a*.^20^ (3) A ∼0.2 ns component, which has a maximum at 670 nm and coincides with the maximum of the static Chl *a Q*_*y*_ absorbance band. This long-lived component is therefore assigned to the ground state recovery of excited Chl *a*, in accordance with previous studies on the cyt *b*_6_*f* complex.^4^ This time component coincides with the previously reported 194 ps mono-exponential component from dissolved diffraction grade cyt *b*_6_*f* crystals (*i.e.* ultra-pure cyt *b*_6_*f* preparation).^4^ The same referred study showed that an extra 6.5 ps component was found to be characteristic of the conventional preparations (*i.e.* with Chl *a*/cyt *f* ratio > 1) and was assigned to the Chl *a* contaminant. Accordingly, we assign our 684 nm band signals (Fig. 4) to be exclusively representative of contaminant Chl *a*. On the other hand, we assign our 670 nm band signal exclusively to the cyt *b*_6_*f*-bound Chl *a*. The work by Dashdorj *et al.* also indicates the presence of a small 5.5 ns component (∼10%), which is potentially assigned to both integral and contaminant Chl *a*. This long-lived signal, if present, would, however, be incorporated in our 0.2 ns component, as our temporal window of 50 ps would not allow us to distinguish between them. It is important to note that this reported 5.5 ns component, because it is assigned to both integral and contaminant Chl *a*, cannot be used to unambiguously distinguish between the two Chl *a* types. However, the 684 nm band signals, because they are exclusively assigned to contaminant Chl *a*, are distinct markers.

In order to verify these assignments, the sample was exposed to the air. Exposing solubilized and unprotected Chl *a* to oxygen is expected to lead to the formation of singlet oxygens and to the subsequent degradation of the nearby molecules, including the unprotected Chl *a* molecule itself. Indeed, it has been shown that solubilized Chl *a* molecules are about 130 times less stable than those embedded in cyt *b*_6_*f*.^4^ The higher stability of integrant Chl *a* is expected to be provided by the nearby carotene molecules in cyt *b*_6_*f*. Therefore, if degradation occurs, it is expected to affect primarily the contaminant Chl *a* molecules. The experiment and data processing are thus repeated on the same cyt *b*_6_*f* preparation, but under aerobic conditions. The sample is left under aerobic conditions for 20 minutes before the start of the experiment. During this short time, we assume that only contaminant Chl *a* molecules are affected by the presence of oxygen, and that the cyt *b*_6_*f* complexes remain otherwise intact, as indicated by the absorption spectrum in Fig. 1. The GF (not shown) and DAS (Fig. 4) result in a minimum of three exponential decay components that are virtually identical in shape and in timings (but not amplitude, as discussed subsequently), within the error margin of the analysis, to those of the anaerobic case. The presence of dissolved oxygen is indeed not expected to affect the ultrafast dynamics of the complex (*i.e.* same spectral shape and decay components). However, under aerobic conditions, the amplitude of both the ∼90 fs and ∼8.9 ps components, both peaking at 684 nm, lowers by 12% compared to the anaerobic case, while the ∼0.2 ns component peaking at 670 nm remains identical. Furthermore, it can be seen in the static absorbance spectra (Fig. 1) that the aerobic Chl *a Q*_*y*_ band is also reduced compared to the initial anaerobic spectrum. This slight decrease in static absorption reflects the degradation of some Chl *a* molecules. This degradation directly coincides with the decrease in the 684 nm transient signals (∼90 fs and ∼8.9 ps components). This correspondence confirms that degradation solely affects the 684 nm signal, which is then exclusively assigned to contaminant Chl *a*. A longer exposure is expected to lead to the degradation of the Chl *a* contaminant to a greater extent. However, nothing guarantees that only contaminant Chl *a* would be affected. And without an objective measure of the impact of oxygen on cyt *b*_6_*f*, the interpretation of the data would become even more complex. Here, we assume that cyt *b*_6_*f* remains intact during this short exposure time.

Concerning the nature of the 684 nm band, it could coincide with the SE and the fluorescence signal from Chl *a*, which are expected to be red-shifted with respect to the static absorbance spectrum.^20,21^ A similar signal at 684 nm can actually be inferred from the transient isotropic pump–probe Chl *a* data (data not shown) as well as from the fluorescence spectra from a previous cyt *b*_6_*f* report.^15^ But in these studies, the 684-nm signal is only present as a faint shoulder to the main 670 nm band, which makes it hardly distinguishable. The present use of parallel polarization fs-spectroscopy greatly improves the resolution of this 684-nm signal and enables its unobstructed spectral and temporal monitoring.

**Fig. 5:**
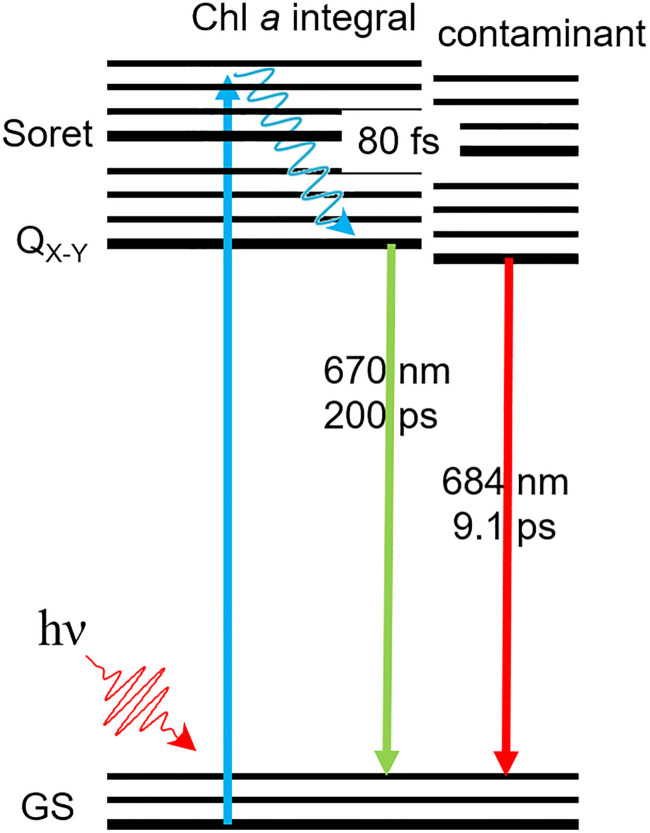
Jablonsky diagram for the integral and contaminant Chl *a*. After Soret-band activation (*hν*), the excited Chl *a* molecules rapidly relax to the lowest *Q*_*y*_ state within 80 fs. While the integral Chl *a* decay back to the ground state (GS) in 200 ps and are marked by a 670-nm signal, the contaminant Chl *a* decay in 9.1 ps and are marked by a 684-nm signal. Note that no evidence is given for any triplet state formation.

Interestingly, we do not monitor any significant Chl *a* triplet state signal. While Chl *a* molecules integral to cyt *b*_6_*f* are not expected to undergo intersystem crossing,^4^ photo-excited Chl *a* monomers are expected to form a long-lived triplet state with an efficiency of ∼64%.^22,23^ The triplet state signal from contaminant Chl *a* could then be expected to contribute to the 670 nm band bleach in Fig. 4. However, the similarity between the 0.2 ns DAS anaerobic (intact) and aerobic (after oxygen-mediated degradation of contaminant Chl *a*), shown in Fig. 4, indicates that contaminant Chl *a* molecules do not significantly contribute to that signal. In light of these conclusions, we offer a tentative Jablonsky diagram in Fig. 5. Because we do not monitor any phosphorescence, contaminant Chl *a* must therefore have a triplet formation yield much lower compared to solvated Chl *a* monomers. Unbound Chl *a* monomers are indeed expected to be washed away during the sample purification process. Hence, we do not expect any unbound Chl *a* monomers. It is however expected that contaminant Chl *a* molecules adsorb to the surface of the cyt *b*_6_*f* complexes. It is therefore assumed that adsorption provides a highly effective deactivation mechanism, which hinders triplet formation. This deactivation route does not prohibit entirely the formation of triplet states (given the long-term degradation monitored), but it is effective enough for the triplet state to not be discernable in our transient experiments.

## Conclusions

In summary, we demonstrate that, although it is impossible to spectrally disentangle the contribution from cyt *b*_6_*f*-bound to contaminant Chl *a* using steady state spectroscopy, time resolved spectroscopy enables such distinction. On the one hand, the excited cyt-embedded Chl *a* is characterized by a single 670 nm band signal that decays mono-exponentially with a decay time of ∼0.2 ns. On the other hand, contaminant Chl *a* molecules give rise to an extra 684 nm band that appears and decays mono-exponentially in ∼85 fs and ∼9 ps, respectively. The assignment of the latter signals to unbound (thus unprotected) Chl *a* is further confirmed by the signal's sensitivity to oxygen. The unambiguous monitoring of contaminant Chl *a* is here made possible by the careful use of parallel polarization between the pump and the probe. This polarization scheme enhances the 684-nm signal, which, based on its lifetime, is taken as a marker of unbound Chl *a* molecules in cyt *b*_6_*f* preparations.

## Conflicts of interest

The authors declare no competing financial interests.

## Abbreviations

Car: β-Carotene
Chl *a*: Chlorophyll *a*
cyt: Cytochrome
DAS: Decay associated spectrum
GF: Global fit
PAGE: Polyacrylamide gel electrophoresis
SE: Stimulated emission
SVD: Singular value decomposition
SDS: Sodium dodecyl sulfate
UDM: Undecyl maltoside

## Supplementary Material

CP-028-D5CP03433G-s001

## Data Availability

The spectroscopic data (transient UV-vis) pertaining to our work entitled “Spectral and temporal differentiation between integral and contaminant chlorophyll *a* in the cytochrome *b*_6_*f* complex” is classified as nonspecific and suitable for general or institutional repository. It is currently stored in local university owned servers, as per the project's data management plan. More explicitly, the data consists of static spectra (vector – wavelength) and transient spectra (matrices – time X wavelength) in MATLAB data files, which will be shared directly upon request. The data supporting this article have been included as part of the supplementary information (SI). The SI includes a deconvolution of the *b*_6_*f* complex static absorption spectra in function of its Chl *a* and Car contributions. The SI also includes calculation of the expected enhancement of signal monitoring due to the use of parallel polarization between pump and probe. See DOI: https://doi.org/10.1039/d5cp03433g.
