# Supplementary material for: Spectral and temporal differentiation between integral and contaminant chlorophyll a in the cytochrome b6f complex
Source: Phys Chem Chem Phys. 2025 Dec 2;28(2):1142–7. doi: 10.1039/d5cp03433g (PMC12709670; doi:10.1039/d5cp03433g)
Supplement: CP-028-D5CP03433G-s001 [file CP-028-D5CP03433G-s001.pdf]

Supplementary Information (SI) for Physical Chemistry Chemical Physics.
This journal is © the Owner Societies 2025

Spectral and Temporal Differentiation between Integral and Contaminant Chlorophyll *a* in the Cytochrome b_6f Complex

Adrien A. P. Chauvet,[a†] Rachna Agarwal,[b,c] and William A. Cramer[b]*

[a] Laboratoire de Spectroscopie Ultrarapide (LSU), ISIC, Faculté des Sciences de Base and Lausanne Centre for Ultrafast Science (LACUS), Ecole Polytechnique Fédérale de Lausanne (EPFL), Station 6, 1015 Lausanne, Switzerland.
[b] Department of Biological Sciences, Purdue University, West Lafayette, Indiana 47907, United States.
[c] Molecular Biology Division, Bhabha Atomic Research Centre, Mumbai 400 085, India
† Present Addresses: School of Mathematic and Physical Sciences, University of Sheffield, Sheffield S3 7HF (UK)
* Corresponding Author: a.chauvet@sheffield.ac.uk

Supplementary Information

1. Static absorption spectra deconvolution

By centering the pump at 515 nm, the goal is to minimize exciting carotenoid (Car) and the various hemes, while avoiding the chlorophyll (Chl) *a* Q_{X-Y} regions. One can still appreciate that most of the pump photons are absorb by Car, as depicted in Figure S 1.

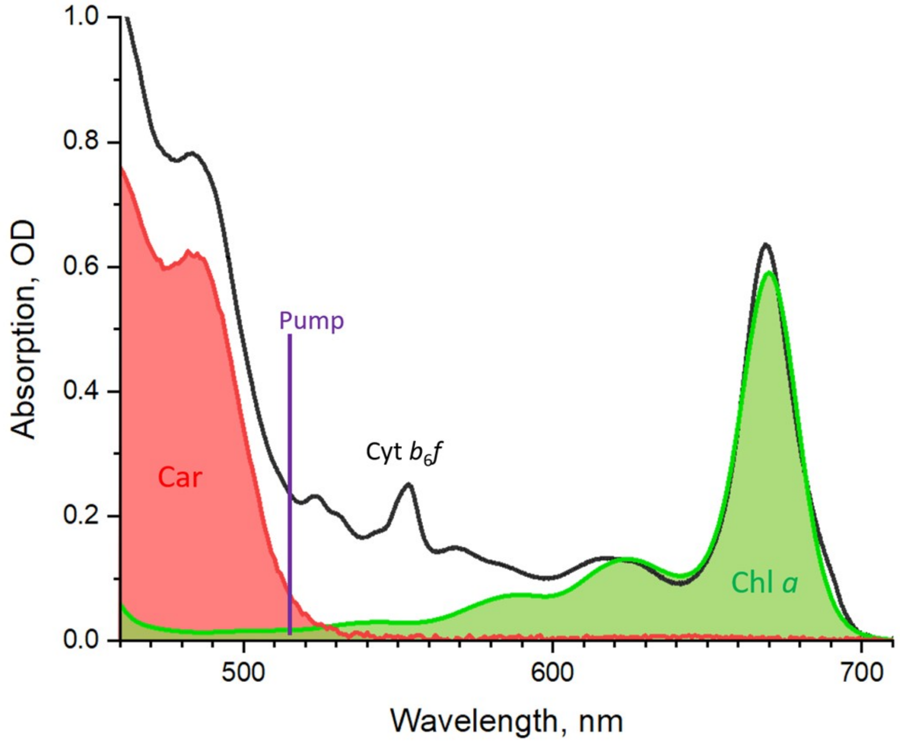

Figure S 1: Absorption of cytochrome (Cyt) b_6f overlayed with an approximate carotenoid (Car) amd chlorophyll a (Chl a) contribution, in relation to the excitation (pump) wavelength at 515 nm.

2. Signal amplitude estimation

This work benefits from a parallel polarization between pump and probe to enhance the monitoring of the Chl *a* signal. The goal is to estimate how amplified is the monitoring of transient signal in a parallel configuration ($S_=$) with respect to signals monitored in a typical magic angle configuration (S_{MA}):

$$\frac{S_=}{S_{MA}}\#(1)$$

Assuming an anisotropy value of 0.4, we have:

$$\frac{(S_= - S_\perp)}{S_= + 2S_\perp} = 0.4\#(2)$$

With $S_\perp$ being the signal monitored at perpendicular polarization.

The later equation can be rearranged as:

$$S_= = 3S_\perp\#(3)$$

which show that signals measured at parallel polarization are expected to be three times larger than those measured at perpendicular polarization.

Furthermore, we have:

$$S_{MA} = \frac{(S_= + 2S_\perp)}{3}\#(4)$$

Which, assuming $S_= = 3S_\perp$, can be written as:

$$S_{MA} = \frac{(3S_\perp + 2S_\perp)}{3} = \frac{5}{3}S_\perp\#(5)$$

Finally, combining equations (3) and (5), we have

$$\frac{S_=}{S_{MA}} = \frac{9}{5} = 1.8\#(6)$$

By selecting a parallel pump-probe configuration, we can expect an enhancement factor of 1.8, with respect to usual magic angel measurements. This enhancement is significant and allows us to disentangle the otherwise buried ~684 nm Chl *a* signal.
